# The Effects of Age, Exposure History and Malaria Infection on the Susceptibility of *Anopheles* Mosquitoes to Low Concentrations of Pyrethroid

**DOI:** 10.1371/journal.pone.0024968

**Published:** 2011-09-22

**Authors:** Katey D. Glunt, Matthew B. Thomas, Andrew F. Read

**Affiliations:** 1 Center for Infectious Disease Dynamics, The Pennsylvania State University, University Park, Pennsylvania, United States of America; 2 Department of Biology, The Pennsylvania State University, University Park, Pennsylvania, United States of America; 3 Department of Entomology, The Pennsylvania State University, University Park, Pennsylvania, United States of America; 4 Fogarty International Center, National Institutes of Health, Bethesda, Maryland, United States of America; Institut Pasteur, France

## Abstract

Chemical insecticides are critical components of malaria control programs. Their ability to eliminate huge numbers of mosquitoes allows them to swiftly interrupt disease transmission, but that lethality also imposes immense selection for insecticide resistance. Targeting control at the small portion of the mosquito population actually responsible for transmitting malaria parasites to humans would reduce selection for resistance, yet maintain effective malaria control. Here, we ask whether simply lowering the concentration of the active ingredient in insecticide formulations could preferentially kill mosquitoes infected with malaria and/or those that are potentially infectious, namely, old mosquitoes. Using modified WHO resistance-monitoring assays, we exposed uninfected *Anopheles stephensi* females to low concentrations of the pyrethroid permethrin at days 4, 8, 12, and 16 days post-emergence and monitored survival for at least 30 days to evaluate the immediate and long-term effects of repeated exposure as mosquitoes aged. We also exposed *Plasmodium chabaudi-* and *P. yoelii*-infected *An. stephensi* females. Permethrin exposure did not consistently increase mosquito susceptibility to subsequent insecticide exposure, though older mosquitoes were more susceptible. A blood meal slightly improved survival after insecticide exposure; malaria infection did not detectably increase insecticide susceptibility. Exposure to low concentrations over successive feeding cycles substantially altered cohort age-structure. Our data suggest the possibility that, where high insecticide coverage can be achieved, low concentration formulations have the capacity to reduce disease transmission without the massive selection for resistance imposed by current practice.

## Introduction

Malaria control programs make extensive use of insecticides to decimate mosquito populations. This very effectively interrupts disease transmission, but necessarily imposes immense selection for insecticide resistance in the targeted mosquito populations. Once resistance arises or migrates into an area, it can spread very rapidly, undermining one of the most effective approaches to malaria control [1], [2], [3], [4], [5], [6], [7].

Attempts to retard the evolution of resistance in mosquito populations typically involve alternating insecticide classes, in effect creating a mosaic of compounds with contrasting modes of action in either time or space. These resistance management strategies assume that resistance to one class of molecule will not be protective against a different class and that mosquitoes pushed to deal with one will have to sacrifice their ability to deal with another [e.g.], [ 8,9]. Cross-resistance poses a significant challenge to these strategies, particularly because only four classes of insecticide involving only two modes of action are approved for public health use [10], [11].

Less-widely utilized resistance-management methods rely on more judicious pesticide application, limiting the time or spatial distribution of spraying to, for example, one season or area [4], [8], [10], [12], [13], [14], [15], [16], [17]. Reducing the proportion of the mosquito population that encounters insecticide limits the relative advantage of resistance and, thus, slows its spread.

A different way of reducing the proportion of the mosquito population experiencing insecticide selection has recently been suggested [18], [ see also], [ 19,20]. Females that feed on an infected host must survive the 10–14 day development period of the parasite before becoming infectious. Female anophelines have high daily mortality rates [21], [22] and have heightened risk of death during blood feeds [23], [24], so that few females live long enough to become vectors even without exposure to control measures. If those few potentially infectious mosquitoes could be selectively removed, malaria control could be achieved without intense selection for resistance. Exclusively targeting the older and, ideally, only the older infected females could therefore achieve malaria control without imposing strong selection for resistance. Younger mosquitoes, which constitute the bulk of the population, would continue to live and reproduce. Indeed, if there are fitness costs to resistance experienced by all individuals, late-life acting insecticides might not impose any net selection for resistance since the benefits of resistance would be experienced by only the few mosquitoes still breeding in late life [18].

The technical challenge is to find a way to selectively eliminate old, potentially infectious females. Here we test the idea [18] that this might be achievable with existing insecticides applied at concentrations lower than those currently recommended. Insecticides are usually deployed with the aim of killing all mosquitoes on contact, and so are applied at concentrations likely to overwhelm individual variation in susceptibility. There are three reasons for thinking that lower concentrations might be disproportionately effective against the mosquitoes responsible for malaria transmission.

First, older mosquitoes from a variety of important vector species are more susceptible than younger mosquitoes to a number of currently-used chemicals. For example, carboxylesterase-based detoxification of malathion in *An. stephensi* and *An. gambiae* slows as mosquitoes age, and even mosquitoes that are malathion-resistant at emergence become increasingly susceptible with age [25]. Susceptibility to permethrin also increases with age in those species [26], as does susceptibility to lambda-cyhalothrin in *An. funestus* [27]. Age-specific susceptibility to DDT has also been reported in *An. gambiae* s.l. [28], [29] and in *An. arabiensis* selected for resistance over 16 generations [30]. Recent work with *An. gambiae* also detected increased susceptibility to bendiocarb in 14-day old mosquitoes [29]. Thus, concentrations of active ingredient too low to significantly impact younger mosquitoes could remove a substantial number of older mosquitoes from the population.

A second reason that lower doses might selectively remove older mosquitoes is that there could be cumulative effects of repeated exposure to normally sub-lethal concentrations of insecticides. In nature, mosquitoes are exposed to insecticides on bednets or walls when they attempt to blood feed on a human. Where insecticide coverage is high, and insecticide resistance driven by public health applications, mosquitoes surviving a first exposure will likely encounter insecticides in subsequent feeding cycles as well. Could one sub-lethal exposure increase susceptibility to the next? Hodjati and Curtis (1999) found that a brief pre-exposure to a low dose of permethrin could increase mortality from a second exposure 24 hours later. If this held true for multiple exposures over successive feeding cycles, older mosquitoes could be effectively targeted by lower doses of insecticide. We note, however, that things could actually go the other way: sub-lethal insecticide exposure can induce the production of detoxification enzymes and, therefore, might decrease the susceptibility of older mosquitoes [31].

A third reason why lower insecticide concentrations might selectively kill the mosquitoes responsible for malaria transmission is that these individuals carry malaria parasites. Harboring *Plasmodium* sp. could impose a metabolic stress on mosquitoes [31], [32], [33], rendering them less able to tolerate insecticide exposure. If so, low doses could be even more effective at interfering with malaria transmission without imposing intense selection for insecticide resistance on the entire mosquito population. The survival impact of malaria infection is not well-defined for anopheline mosquitoes, often varying among vector-parasite combinations [34], [35], [36]. We are unaware of any work on the consequences of malaria infection on mosquito susceptibility to insecticides, though infection with entomopathogenic fungi has been shown to increase susceptibility [37].

If lower doses of already-approved public health insecticides could deliver effective malaria control with less selection for resistance (what we call the ‘dilution solution’), the benefits would extend beyond the resistance management: reducing the quantity of active ingredient applied around and within dwellings will reduce costs and any health and environmental impacts. Using permethrin, a pyrethroid widely used for malaria control, we therefore asked whether insecticide concentrations lower than the standard WHO resistance-discriminating dose would: (1) disproportionately kill old, potentially-dangerous mosquitoes, (2) become increasingly lethal with repeated exposure, and/or (3) selectively remove malaria-infected mosquitoes.

## Materials and Methods

### Experimental overview

**Figure 1:**
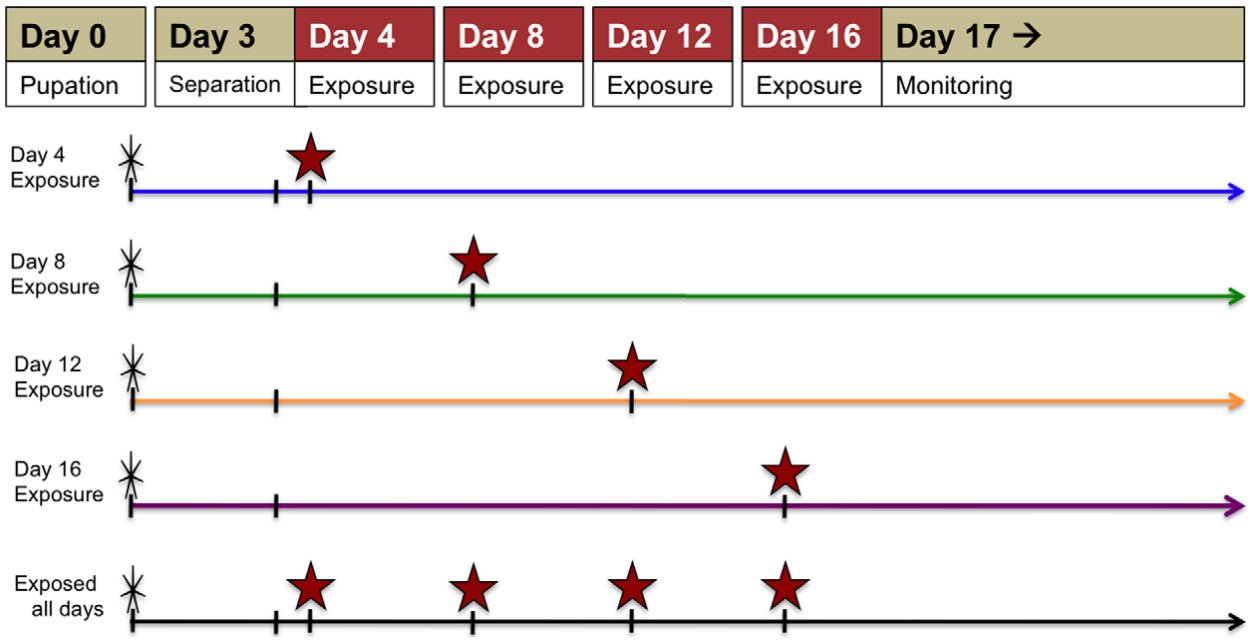
Exposure scheme for exposure-history experiments. A single cohort of adult female mosquitoes was split into five groups three days after emergence. Insecticide-naïve and previously-exposed females were exposed to various concentrations of permethrin on days indicated by the red stars. Knockdown was assessed at the end of each 1 h exposure and survival assessed 24 h later. Survival of all mosquitoes was monitored daily for at least 30 days.

**Figure 2:**
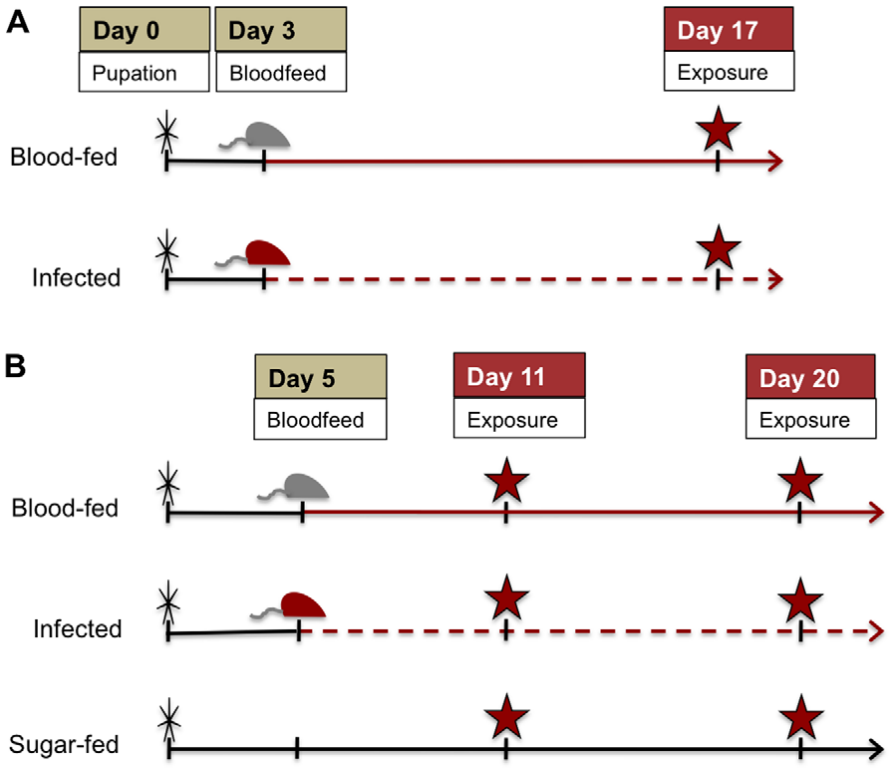
Schedules for malaria experiments. For each experiment, female mosquitoes from a single cohort were divided into treatment groups. (A) In both *P. chabaudi* experiments, females were bloodfed at 3–5 days post-emergence from either uninfected or infected mice and then exposed to permethrin 14 days later. (B) In the *P. yoelii* experiment, females were fed uninfected or infected blood five days after emergence, or were only given sugar. These groups were exposed to permethrin twice, as indicated by the red stars. Knockdown was assessed at the end of each 1 h exposure and survival assessed 24 h later.

We conducted two kinds of experiments with doses of permethrin low enough to leave young mosquitoes alive: (i) exposure-history experiments, aimed at evaluating the possibly distinct effects of age-at-exposure vs. previous contact on survival, and (ii) malaria experiments. We conducted two of the former and three of the latter. Experimental designs and timing of insecticide exposures are shown in Figs. 1 and 2. In the exposure-history experiments (Fig. 1), we exposed insecticide-naïve and previously-exposed females at 4 different time points designed to mimic feeding bouts and monitored survival for at least 30 days. In the malaria experiments, females were infected with *Plasmodium chabaudi* (Fig. 2A) or *P. yoelii* (Fig. 2B). We exposed *P. chabaudi*-infected females to permethrin only once, at 14 days post-infection (Fig. 2A). Based on the extrinsic incubation period of the parasite, most infected mosquitoes would be infectious on this day [38]. In the *P. yoelii* experiment, we scheduled the exposures based on feeding cycle length and malaria infection status to cover the pre-infectious (oocyst) stage (day 6 pi), or when most were expected to be infectious (day 15 pi) (Fig. 2B).

### Mosquito rearing and maintenance

*Anopheles stephensi* LISTON with no known previous exposure to insecticides were obtained from NIH and cultured at Penn State since 2008 under standard insectary conditions of 27±1°C, 65±5% RH, and a 12 L∶12 D photoperiod. Eggs were placed in plastic trays (25 cm×25 cm×7 cm) filled with 1.5 L of distilled water. To reduce variation in adult size at emergence, larvae were reared at a fixed density of 400 per tray. Larvae were fed Liquifry for five days and then ground TetraFin fish flakes. From approximately two weeks after egg hatch, we collected pupae daily and placed them in emergence cages. The adults that emerged were fed *ad libitum* on a 10% glucose solution supplemented with 0.05% paraaminobenzoic acid (PABA).

Experimental mosquitoes were maintained at 25±2.3°C, 90±5% RH before and after insecticide exposure or, because *P. yoelii* has a lower optimum temperature for development [39], at 22±1°C, 90±5% RH during the *P. yoelii* experiment. To more closely regulate the mosquitoes' environment during the second exposure-history experiment, a constant-temperature incubator was used; it ran at 27±1.5°C, 90±5% RH. Glucose/PABA was available *ad libitum*.

### Definition of age

The WHO specifies that resistance monitoring assays should be conducted using mosquitoes 24–48 h post-emergence [40]. With this in mind, we defined the age of our experimental mosquitoes based on time post-emergence and established the population with only a 2-day range of ages. For example, if pupae were added to a cage on day 0, the adults in that cage the next day were considered to be one day old. The pupal bowl was then removed from the cage on day 2, when the mosquitoes in the cage were 1–2 days old.

### Permethrin exposures

Filter paper sheets were impregnated with technical-grade permethrin (ChemService, West Chester, PA) or control solution, according to WHO protocol [11], at least 24 hours prior to use. Acetone acted as the solvent for the insecticide; silicon oil (Dow Corning 554) served as the carrier. We calculated concentrations based on the mg of active ingredient per unit of oil [11].

The WHO established the discriminating dose for testing permethrin resistance at 0.75%. Our choice of test concentrations was identified from range-finding experiments and included concentrations killing only a minority of young mosquitoes. In exposure-history experiment 1, we used permethrin concentrations of 0, 0.06, 0.08, and 0.1%. In the second exposure-history experiment, we replaced the lowest concentration (0.06%) for one expected to generate moderately high lethality (0.25%). We used 0.085 and 0.1% in *P. chabaudi* experiment 1, and 0.02, 0.04, 0.05, 0.06, and 0.08% in experiment 2; we exposed mosquitoes to 0.08 and 0.25% in the *P. yoelii* experiment.

In all experiments, insecticide exposure followed the WHO insecticide-resistance assay protocol [40] with a few modifications. Under standard insectary conditions, we transferred female mosquitoes by mouth aspirator to plain paper-lined holding tubes for a brief acclimation before blowing them gently into the connecting exposure tubes. At the end of the 60-min exposure period, we scored mosquitoes for knockdown (see below) and then gently blew mosquitoes into mesh-covered paper cups, provided them with glucose, and monitored their subsequent survival. For the exposure-history experiments, adult mosquitoes were kept at similar densities by allocating 2–4 day old females into treatment groups or replicates before the experiments began. In the first exposure-history experiment, we started with groups of twenty 2–3 day old females. The second exposure-history experiment started with groups of 15.

On each exposure day, we exposed batches of mosquitoes in three experimental blocks, with each treatment group represented at least once in each block. In both exposure-history experiments, with 4 concentrations of permethrin and 5 treatments for each group, there were 60 cups total. In the *P. chabaudi* experiments, we used 2 treatments and either 3 or 6 concentrations of permethrin, resulting in either 18 or 36 cups of mosquitoes. In both exposures of the *P. yoelii* experiment, there were 3 treatments, 3 concentrations of permethrin, and 3 replicates per experimental group, for a total of 27 cups. We exposed 4 batches of mosquitoes in exposure 1 of the *P. yoelii* experiment, but fewer mosquitoes were available for *P. yoelii* exposure 2, so we exposed 3 batches instead.

### Malaria infections

Female experimental mice (C57 Bl/6) were infected with 10^6^ parasites of the rodent malaria, *P. chabaudi* (clone ER, from the WHO Registry of Standard Malaria Parasites, University of Edinburgh, UK) or 10^5^ *P. yoelii* (clone 17XNL, from the WHO Registry of Standard Malaria Parasites, University of Edinburgh, UK). The following reagent was obtained through the MR4 (MRA-886 *P. yoelii* 17XNL), deposited by New York University School of Medicine. For *P. chabaudi* infections, mosquito blood feeds took place on days 12 or 13 post-infection, when all mice had gametocytaemia >0.1%; *P. yoelii* infections took place on day 4 pi. Mosquitoes in control cages fed on the same number of uninfected mice. All mice were anesthetized prior to mosquito feeds using a Xylazine∶Ketamine (0.15∶1) mix at 0.1 ml/10 grams body weight i.p., and all efforts were made to minimize suffering. This study was carried out in strict accordance with the recommendations in the Guide for the Care and Use of Laboratory Animals of the National Institutes of Health. The protocol was approved by the Animal Care and Use Committee of the Pennsylvania State University (Permit Number: 27452).

Mosquitoes exposed to *P. yoelii* were placed at 22° immediately after the blood feed. Eight days after an infectious blood meal, parasite burdens in mosquitoes were assessed by dissecting 25 mosquitoes in phosphate-buffered saline (PBS).

### Response variables

At the end of the 1 h exposure period, before transferring the mosquitoes to cups, we scored mosquitoes that did not fly after gentle tapping and rotation of the exposure tube as knocked down (*Knockdown after 1 hr*). One day following each exposure, we determined the proportion of exposed mosquitoes remaining alive in a cup (*24 h survival*). For the at least 30 days of the repeated-exposure experiments, we determined the proportion of mosquitoes remaining alive each day (*Cumulative survival*).

### Statistical analysis

Statistical analyses were performed in SPSS v.18 (PASW 18.0). Data on 1 h knockdown and survival 24 h after exposure were analyzed as Generalized Linear Models using a binomial error distribution and logit link function, with factor categories in descending order. We fit maximal models with interaction terms first and sequentially removed non-significant terms, beginning with highest order interactions. Independent variables included *permethrin concentration*, *age at exposure* (4, 8, 12, 16 days), *number of exposures* (1, 2, 3 or 4), *infection status* (blood meal malaria-infected or not), and *feeding* (sugar-only, uninfected blood, malaria-infected blood). Least Significant Difference post-hoc pairwise comparison tests were used. Kaplan-Meier survival analyses were conducted using cumulative survival data, stratifying by permethrin concentration. Factor levels were compared first for each stratum and then, to distinguish differences among groups within concentrations, pairwise for each stratum. To investigate cumulative effects of exposure, we used log-rank tests to compare survival distributions. Where we pooled data from across experiments (the two exposure-history experiments, the two *P. chabaudi* experiments), we fitted ‘experiment’ in the models. In most cases, ‘experiment’ was significant, indicating differences in mean survival between the two experiments. Since these differences are uninteresting, we do not report them below. In some cases, there were significant interactions between ‘experiment’ and other factors being tested, but in all cases these represented minor differences in magnitude, not qualitative differences between experiments.

## Results

### Exposure history

**Figure 3:**
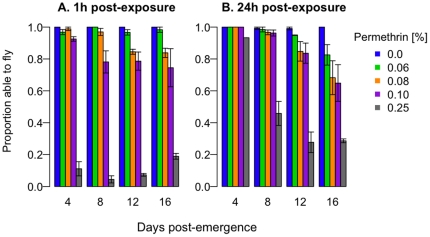
The effect of age on susceptibility to low permethrin concentrations. Groups of female mosquitoes were exposed to permethrin once, at 4, 8, 12, or 16 days post-emergence. At the end of each hour-long exposure, we recorded the number of mosquitoes able to fly (A) and, 24 hours later, the number alive (B). As mosquitoes get older, they are less likely to survive permethrin exposure, especially at higher concentrations. Bars indicate the mean ±1 SE.

For mosquitoes exposed to permethrin only once, both age at exposure and permethrin concentration affected susceptibility to insecticides (Fig. 3A, Knockdown: *permethrin*, χ^2^_df = 3_ = 380.5, p<0.001; *age at exposure*, χ^2^_df = 3_ = 199.7, p<0.001; *permethrin*×*age*, χ^2^_df = 8_ = 2021.8, p<0.001; Fig. 3B, Survival: *permethrin*: χ^2^_df = 3_ = 3.9, p = 0.28; *age at exposure*: χ^2^_df = 3_ = 43.9, p<0.001; *permethrin*×*age*: x^2^_df = 7_ = 16.84, p = 0.02). More mosquitoes were knocked down or killed at higher permethrin concentrations and, at a given concentration, older mosquitoes were more susceptible, especially at higher concentrations.

**Figure 4:**
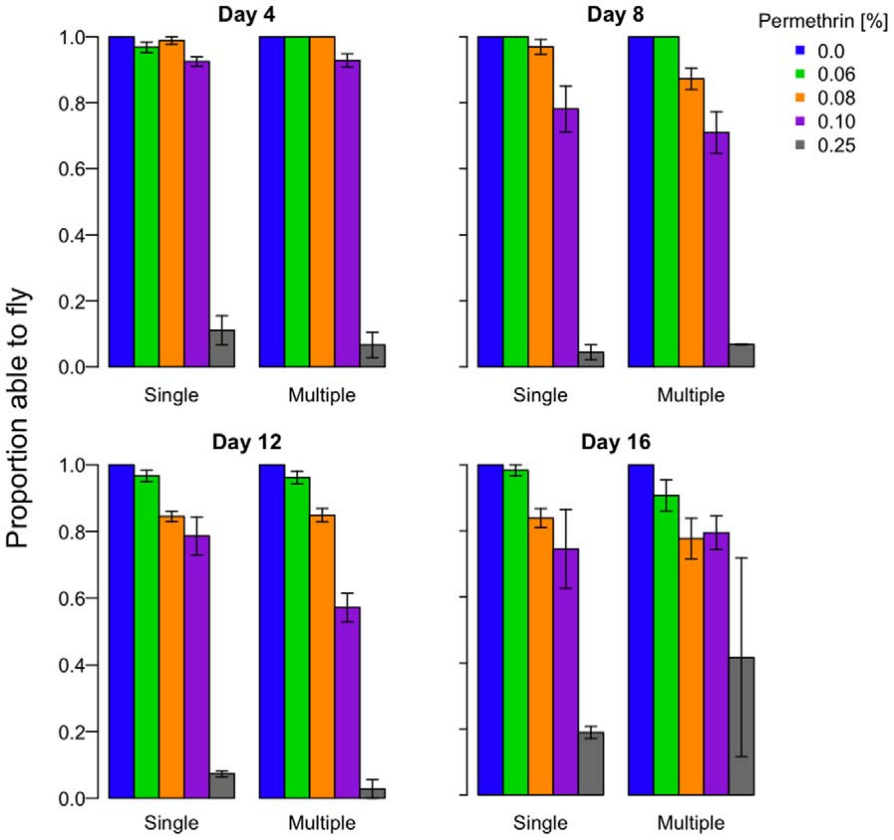
The effects of exposure history on knockdown after 60 min. The 4 panels show the results of the permethrin exposures on days 4, 8, 12, or 16. In each panel, the cluster of bars on the left represent the proportion of insecticide-naïve females able to fly at the end of each exposure. The bars on the right half show the corresponding results for previously-exposed females. In the day 12 panel, for example, multiple exposures had occurred at days 4, 8 and 12, whereas the single exposures occurred on day 12 only. All mosquitoes on day 4 were naïve (see Fig. 1).

**Figure 5:**
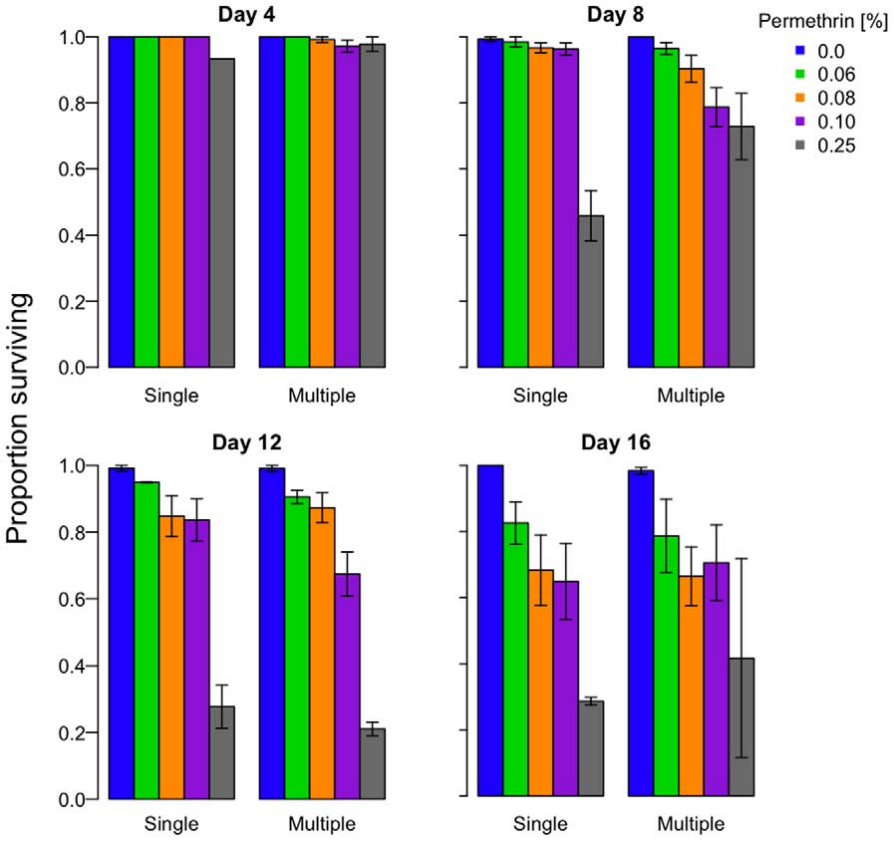
The effects of exposure history on survival after 24 hours. The 4 panels show the results of the permethrin exposures on days 4, 8, 12, or 16. In each panel, the cluster of bars on the left represent the proportion of insecticide-naïve females surviving 24 h after exposure. The bars on the right half show the corresponding results for previously-exposed females. In the day 12 panel, for example, multiple exposures had occurred at days 4, 8 and 12, whereas the single exposures occurred on day 12 only. All mosquitoes on day 4 were naïve (see Fig. 1).

When mosquitoes were exposed to insecticides more than one time, there were situations in which previous exposure affected susceptibility to subsequent permethrin exposures. Sub-lethal exposure at days 4 and 8 increased knockdown rates following exposure at days 8 and 12, respectively, but visual inspection showed that the effect were rather small and did not occur at most concentrations (Fig. 4, compare left and right bars in each panel; *number of exposures*: Day 8 exposure, χ^2^_df = 1_ = 4.8, p = 0.03; Day 12 exposure, χ^2^_df = 1_ = 5.7, p = 0.02). Knockdown rates at day 16 were unaffected by exposure history (Day 16 exposure, χ^2^_df = 1_ = 1.4, p = 0.24). Previous exposure affected 24 h survival rates for exposures on only day 8 (Fig. 5), an effect that depended on permethrin concentration and had contrasting effects at the intermediate and highest concentrations (*number of exposures* (here once or none): χ^2^_df = 1_ = 2.8, p = 0.1; *permethrin×number of exposures* interaction: χ^2^_df = 3_ = 19.8, p<0.001). At 12 days post-emergence, when previously-exposed mosquitoes had already experienced 2 contacts with permethrin, previously-exposed females were marginally more susceptible (Exposure 3, *number of exposures*: χ^2^_df = 1_ = 4.0, p = 0.05). At 16 days post-emergence, when previously-exposed mosquitoes had already experienced 3 contacts with permethrin, previously-exposed and insecticide-naïve females were equally susceptible (Exposure 4, *number of exposures*: χ^2^_df = 1_ = 0.0, p = 0.99). Overall, these results show that the mosquitoes that survive insecticide exposure can be more vulnerable when exposed again some days later, though the effect was relatively minor, not cumulative, was not systematically dependent on concentration and, critically, was not detectable among the older mosquitoes. In contrast, age at exposure had a substantial influence on susceptibility (Fig. 3B; *Age at exposure*: χ^2^_df = 3_ = 43.9, p<0.001.

**Figure 6:**
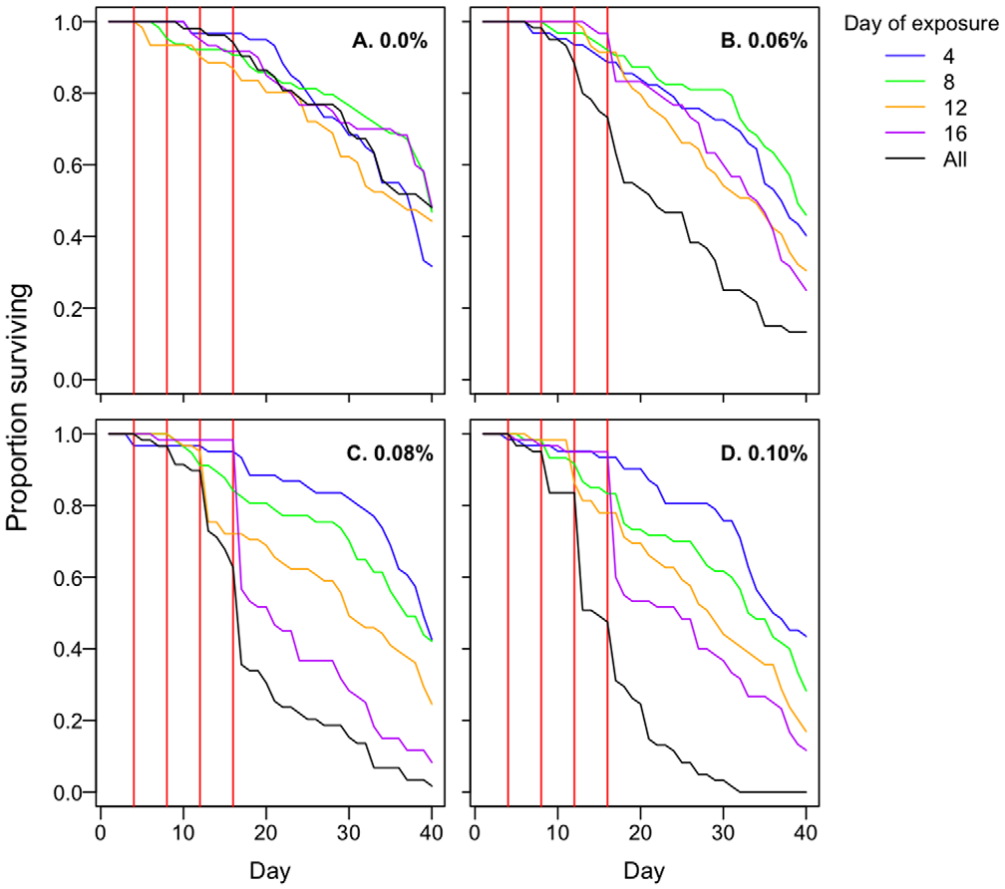
Kaplan-Meier survival curves of mosquitoes exposed to permethrin in exposure-history experiment 1. Panels reflect different permethrin concentrations, as denoted. Red vertical lines show timing of exposures. Plotted data were from the total number of mosquitoes in the 3 replicate cups in each treatment group (see methods), so that n = 60 at the start of each curve. Colors correspond to the timelines in Fig. 1.

**Figure 7:**
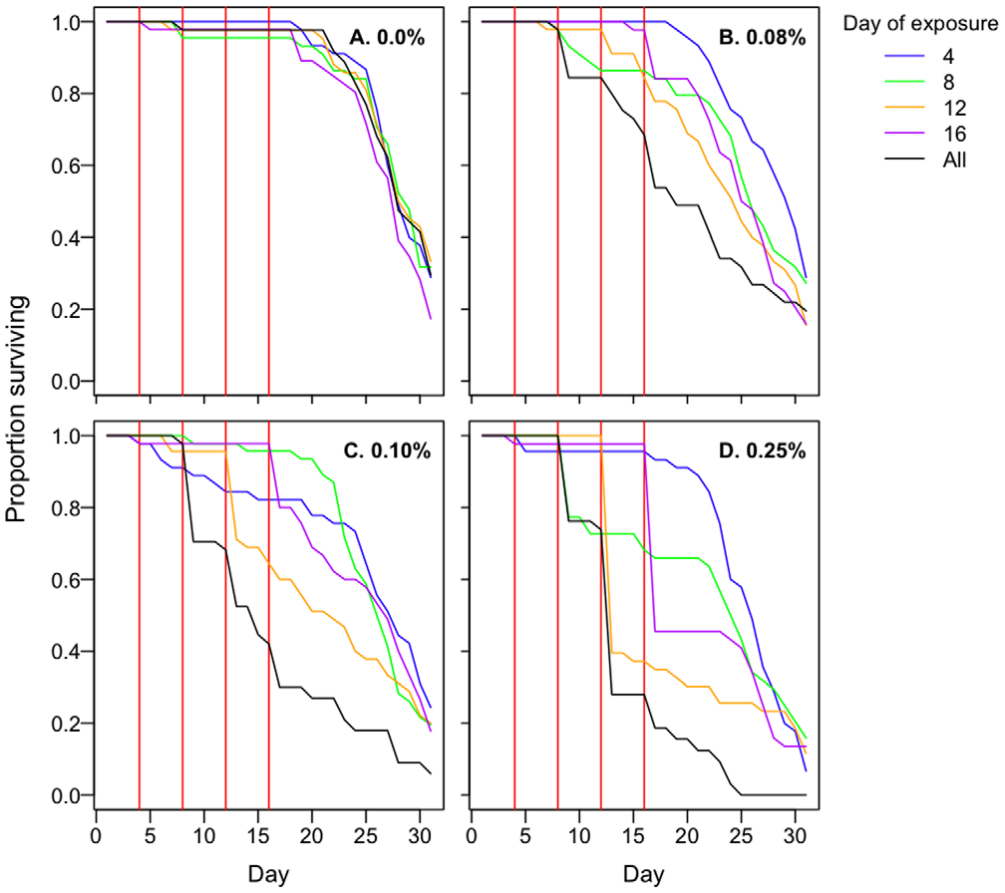
Kaplan-Meier survival curves of mosquitoes exposed to permethrin in exposure-history experiment 2. Panels A–D reflect different concentrations, as denoted. Red vertical lines show timing of exposures. Plotted data were from the total number of mosquitoes in the 3 replicate cups in each treatment group (see methods), so that n = 45 at the start of each curve. Colors correspond to the timelines in Fig. 1.

None of the survival curves from mosquitoes unexposed to permethrin differed, even though one control group experienced 4 experimental manipulations (Fig. 6A, Experiment 1, *0.0%*: χ^2^_df = 4_ = 4.0, p = 0.41; Fig. 7A, Experiment 2, *0.0%*: χ^2^_df = 4_ = 3.30, p = 0.51).

In both exposure-history experiments, overall mosquito survival differed at each permethrin concentration (Fig. 6B–D, Experiment 1, *0.06%*: χ^2^_df = 4_ = 41.9, p<0.001; *0.08%*: χ^2^_df = 4_ = 96.1, p<0.001; *0.1%*: χ^2^_df = 4_ = 131.4, p<0.001; Fig. 7B–D, Experiment 2, *0.08%*: χ^2^_df = 4_ = 12.9, p = 0.01; *0.1%*: χ^2^_df = 4_ = 32.1, p<0.001; *0.25%*: χ^2^_df = 4_ = 51.9, p<0.001). The number of mosquitoes surviving four successive exposures was less than the number surviving just one exposure. This was because each exposure eliminated a fraction of mosquitoes, and that fraction cumulated additively over successive exposures (Fig. 6B–D, Fig. 7C–D; single exposure vs. multiple-exposure groups: Experiment 1, *all concentrations*: p≤0.001; Experiment 2, *0.08%*: *exposed day 4 vs. exposed all days*, χ^2^ = 9.9, p = 0.002; *exposed day 8 vs. exposed all days*, χ^2^ = 3.9, p = 0.05). There were two minor exceptions: the overall survival curve of mosquitoes exposed to 0.08% on all days in the second experiment (Fig. 7B) was not statistically different from that of those exposed only on day 12 or only on day 16 (*exposed day 12 vs. exposed all days*: χ^2^ = 0.91, p = 0.34; *exposed day 16 vs. exposed all days*: χ^2^ = 2.1, p = 0.15).

### Malaria infection

**Figure 8:**
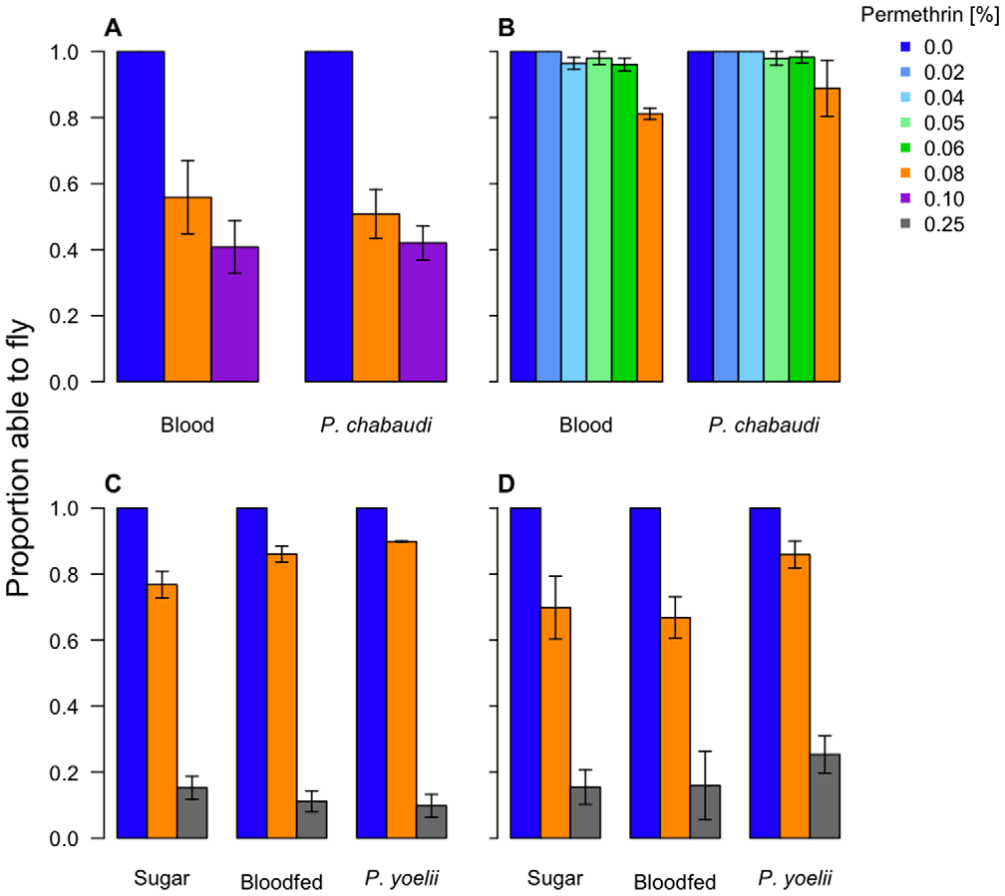
The effects of malaria infection on susceptibility to knockdown. The top 2 panels show the results from *P. chabaudi* experiment 1 (A) and 2 (B). Bars on the left half of each panel are for females fed on uninfected blood; bars on the right are for females fed on infected blood. The bottom two panels show the results from the *P. yoelii* experiments 1 (C) and 2 (D). In each of these 2 panels, the clusters of bars represent, from left to right, the proportion of flying mosquitoes fed on uninfected blood, infected blood, and sugar.

**Figure 9:**
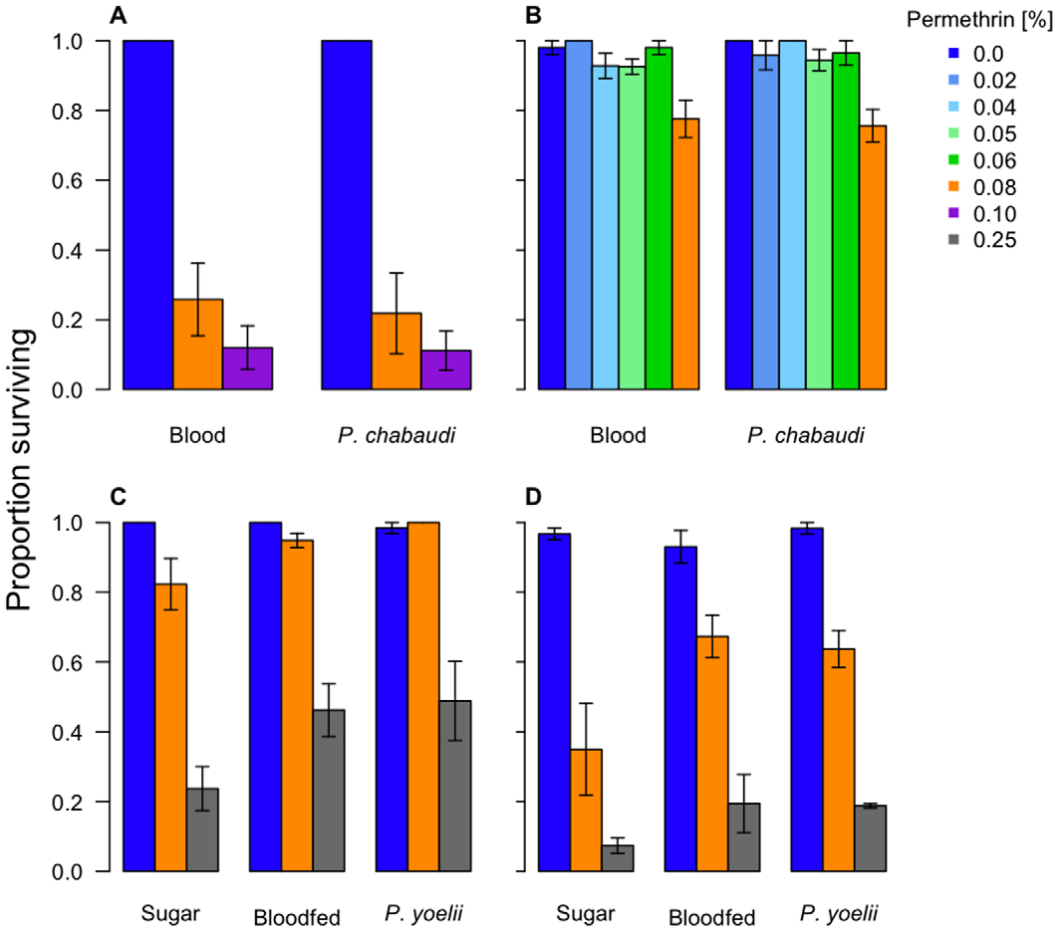
The effects of malaria infection on survival 24 hours after permethrin exposure. The top 2 panels show the results from *P. chabaudi* (A) experiment 1 and (B) 2, with the bars on the left half of each panel reflecting the proportion of uninfected-bloodfed females alive 24 h after exposure. Bars on the right half of each panel represent the females that fed on infected mice. The bottom 2 panels show the results from the *P. yoelii* experiment's (C) exposure 1 and (D) exposure 2. In each of these 2 panels, the clusters of bars represent, from left to right, the proportion of surviving mosquitoes fed on uninfected blood, infected blood, and sugar.

In the first *P. chabaudi* experiment, approximately 40% of females were infected with oocysts (mean burden ± SE = 1.4±0.3 oocysts), while nearly 86% were infected in the second (11.3±3.3 oocysts). Sixty-eight percent of mosquitoes that fed on *P. yoelii*-infected mice developed oocysts (16.0±4.3 oocysts). As expected, mosquitoes were more susceptible to higher permethrin concentrations, regardless of age, blood feeding or infection status (Fig. 8A–D, Fig. 9A–D; in all cases, *permethrin*: p≤0.001).

Regardless of *Plasmodium* species, malaria infection status did not enhance susceptibility to permethrin (Fig. 8, Knockdown: *Infection status*, χ^2^_df = 1_ = 0.05, p = 0.82; Fig. 9, Survival: *Infection status*: χ^2^_df = 1_ = 0.28 p = 0.6). Indeed, late-stage *P. yoelii* infection slightly reduced susceptibility of female mosquitoes to knockdown, relative to sugar- and blood-fed individuals (*P. yoelii*, exposure 2, χ^2^_df = 2_ = 7.9, p = 0.02; Least significant difference, exposure 2, *blood* vs. *infected*: p = 0.01, *sugar* vs. *infected*: p = 0.02, *sugar* vs. *blood*: p = 0.75). Access to a blood meal, rather than the *P. yoelii*-infection status of the blood, had the greatest effect on permethrin susceptibility late in life (Fig. 9C–D, Survival: LSD, in exposures 1 and 2, *sugar* vs. *bloodfed*, *sugar* vs. *infected*: both p<0.001), with blood meals reducing susceptibility to permethrin. Although the *P. yoelii* experiments were not designed to test for an age effect, increased susceptibility to permethrin is apparent in the older mosquitoes (compare Fig. 9C and D).

Together, these data provide no evidence that malaria infection increases the susceptibility of mosquitoes to permethrin. Note that we have here compared the survival of malaria-exposed and non-exposed mosquitoes, not malaria-infected or uninfected mosquitoes. This reduces our power to detect an impact of malaria infection, if it is occurring. Power calculations (not shown) reveal that with the sample sizes and infection rates we had, we could have detected a 25% increase in permethrin-induced death due to malaria. That means that if malaria infection does make mosquitoes more vulnerable to insecticides, and we failed to detect it, the effect is relatively modest.

## Discussion

Refining insecticidal vector control measures to target that small portion of the mosquito population which is actually responsible for infecting humans could considerably reduce the selection for insecticide resistance while still providing effective malaria control [18], [19], [20]. Here, we investigated whether simply lowering the dose of insecticide applied could selectively eliminate the older or malaria-infected proportion of the mosquito population. We found that infection with either species of rodent malaria did not increase mosquito susceptibility to permethrin (Figs. 8, 9). We note that only around two-thirds of the mosquitoes exposed to malaria became infected; the presence of uninfected females among the ‘malaria-infected’ group reduces our power to detect increased vulnerability to permethrin caused by malaria. If an effect of infection on insecticide susceptibility exists, it must be too subtle to be safely exploited in the design of resistance-conscious malaria control programs.

We also found no evidence that previous exposure increased susceptibility to subsequent exposures: mortality rates on the fourth exposure were identical for age-matched mosquitoes experiencing their first exposure (Fig. 5). However, we did find, as have others [25], [26], [27], [28], [29], [30], that older mosquitoes were more susceptible to insecticides (Fig. 3). Permethrin applied at a third of the WHO-recommended resistance-monitoring dose preferentially killed older mosquitoes.

Exposure to insecticides over four-day intervals (designed to mimic feeding cycles) had a marked impact on the age structure of surviving mosquitoes compared to those handled identically but not experiencing insecticides, especially at the higher concentrations we explored (compare Fig. 6A with 6D, and Fig. 7A with 7D). A fraction of the mosquitoes died with each exposure, and the size of that fraction increased with each successive contact with permethrin; by day 20, we had removed more than 75% of the cohort. In nature, most mosquitoes do not live long enough to transmit malaria, so such age-structure-altering approaches to vector control, in further reducing the number of individuals in the older age classes, could be usefully integrated into disease control programs [18], [19], [41], [42], [43].

Control measures that work by altering age-structure also have implications for resistance management [18], [19], [20]. Though an assessment of the consequences of low-dose exposure on fecundity would be required for a full evaluation of the resistance-proofing capabilities of this strategy, targeting the older mosquitoes reduces the selection pressure on the younger majority of the egg-laying population to be resistant to the insecticide [18]. In the absence of sub-lethal fitness effects, it might be possible to use a slightly higher concentration than we used here, so as to kill a slightly greater proportion of younger mosquitoes and thus be sure to eliminate almost all females before they become old enough to transmit malaria. Furthermore, other chemicals that induce age-specific mortality might be more amenable to adaptation for low-dose use in the field. Candidates might include other active ingredients currently approved for malaria control [e.g., 25], insecticides in use in other contexts but currently considered insufficiently lethal to young mosquitoes, as well as novel compounds.

Shifting the age-structure of the mosquito population in this stepwise fashion, so that cumulative mortality by the age mosquitoes become infectious is very high, would require extensive spatial coverage of low-concentration formulations. However, areas where vector control coverage is high are where this application method would be most beneficial for resistance management: when mosquitoes are likely to encounter the insecticide, the pressure for them to be resistant to its effects is greater. Where high coverage cannot be achieved, resistance evolution driven by public health use of insecticides is not a problem.

Selective elimination of older mosquitoes could already be happening in the field. Even partially- and fully-resistant *Anopheles* become more susceptible to insecticide as they age [25], [26], [27], [28], [29], [30]. Bioassays for resistance use young females [29], [40], but where these reveal a high level of resistance in an area, the infected, older mosquitoes might still succumb to exposure. Similarly, as the concentration of insecticide available on a bednet or an indoor-sprayed surface declines with time and becomes less effective against young mosquitoes, malaria control might still be maintained, the low concentrations remaining high enough to kill older mosquitoes. As far as we are aware, this effect has not been explored but could have important implications for determining the persistence of malaria control efficacy of insecticides in real field settings. Indeed, the relationship between resistance and the capacity of *Anopheles* populations to transmit malaria remains to be determined [31].

An objection to managing resistance evolution by applying low doses of insecticide in a field setting is the conventional wisdom that resistance is selected to high frequency via the benefit of partial resistance of heterozygotes that are able to survive low-dose exposures [e.g., 13]. Previous work with free-flying mosquitoes and bednets treated with high and low concentrations of permethrin, however, showed that resistant heterozygotes delayed take off on lower concentrations, extending their contact time and dose and increasing their risk of dying [13], [26]. Additionally, our data show that if low doses are used so that most of the lethality occurs later in life, individuals lacking a resistance allele will suffer greatest fitness consequences after the bulk of their reproduction has occurred. At the same time, given the high daily mortality rate of mosquitoes, the fitness benefit conferred by resistance alleles is experienced by only a small fraction of the population. Thus, if low doses impose selection only late in life, there is in fact very weak selection for resistance [18], [19], [20]. If a fitness cost is associated with a resistance mutation, this can further reduce the selection pressure for genetically-based resistance mechanisms to develop within the population.

There are a number of questions that would need to be answered to evaluate the operational utility of simply using less insecticide to simultaneously control malaria and better manage insecticide resistance. For instance, are there fitness consequences from sub-lethal exposure? Might infection with a human malaria such as *P. falciparum* affect mosquitoes' susceptibility to insecticides? Can appropriate concentrations be consistently applied, given the real-world complexities associated with variation in application? To what extent does concentration affect insecticide persistence in the field? Would communities accept control measures that do not remove nuisance mosquitoes? How would the use of less insecticide affect the commercial drivers for public health insecticides? Under what epidemiological circumstances could this approach work?

Finally, we note that agricultural use of insecticides can be the dominant source of selection for resistance in mosquito populations [44]. In such cases, any resistance management strategy centered on the public health use of insecticides, including rotations, mosaics and age-structure-alterations will have little impact. However, our data suggest that, where high coverage of public health insecticides is achieved and is contributing considerable selection for resistance, it would be worth exploring the potential of low concentration formulations, because they have the capacity to reduce disease transmission without the enormous selection for resistance imposed by current practice. If this could be made to work, existing public health insecticides could have a sustainable future, doing away with the expense of an open-ended insecticide discovery pipeline.

## References

[1] Hargreaves K, Hunt RH, Brooke BD, Mthembu J, Weeto MM (2003). *Anopheles arabiensis* and *An. quadriannulatus* resistance to DDT in South Africa.. Med Vet Entomol.

[2] Hargreaves K, Koekemoer LL, Brooke BD, Hunt RH, Mthembu J (2000). *Anopheles funestus* resistant to pyrethroid insecticides in South Africa.. Med Vet Entomol.

[3] Hemingway J, Penilla RP, Rodriguez AD, James BM, Edge W (1997). Resistance managment strategies in malaria vector control. A large-scale field trial in southern Mexico.. Pestic Sci.

[4] Penilla RP, Rodriguez AD, Hemingway J, Trejo A, Lopez AD (2007). Cytochrome P-450-based resistance mechanism and pyrethroid resistance in the field *Anopheles albimanus* resistance management trial.. Pestic Biochem Physiol.

[5] Kelly-Hope L, Ranson H, Hemingway J (2008). Lessons from the past: managing insecticide resistance in malaria control and eradication programmes.. Lancet Infect Dis.

[6] Ranson H, N'Guessan R, Lines J, Moiroux N, Nkuni Z (2011). Pyrethroid resistance in African anopheline mosquitoes: what are the implications for malaria control?. Trends Parasitol.

[7] Zaim M, Guillet P (2002). Alternative insecticides: an urgent need.. Trends Parasitol.

[8] Curtis CF, Cook LM, Wood RJ (1978). Selection for and against insecticide resistance and possible methods of inhibiting the evolution of resistance in mosquitoes.. Ecol Entomol.

[9] Tabashnik BE (1989). Managing resistance with multiple pesticide tactics: theory, evidence, and recommendations.. J Econ Entomol.

[10] Nauen R (2007). Insecticide resistance in disease vectors of public health importance.. Pest Manag Sci.

[11] WHO (2006). Guidelines for testing mosquito adulticides for indoor residual spraying and treatment of mosquito nets..

[12] Curtis CF (1985). Theoretical models of the use of insecticide mixtures for the management of resistance.. Bull Entomol Res.

[13] Curtis CF (1998). Insecticide treated mosquito nets.. Trop Dr.

[14] Hemingway J, Beaty BJ, Rowland M, Scott TW, Sharp BL (2006). The Innovative Vector Control Consortium: improved control of mosquito-borne diseases.. Trends Parasitol.

[15] Hougard JM, Corbel V, N'Guessan R, Darriet F, Chandre F (2003). Efficacy of mosquito nets treated with insecticide mixtures or mosaics against insecticide resistant *Anopheles gambiae* and *Culex quinquefasciatus* (Diptera: Culicidae) in Cote d'Ivoire.. Bull Entomol Res.

[16] Kolaczinski JH, Curtis CF (2004). Investigation of negative cross-resistance as a resistance-management tool for insecticide-treated nets.. J Med Entomol.

[17] Sharp BL, Ridl FC, Govender D, Kuklinski J, Kleinschmidt I (2007). Malaria vector control by indoor residual insecticide spraying on the tropical island of Bioko, Equatorial Guinea.. Malar J.

[18] Read AF, Lynch PA, Thomas MB (2009). How to make evolution-proof insecticides for malaria control.. PLoS Biol.

[19] Koella JC, Lynch PA, Thomas MB, Read AF (2009). Towards evolution-proof malaria control with insecticides.. Evol Appl.

[20] Gourley SA, Liu R, Wu J (2011). Slowing the evolution of insecticide resistance in mosquitoes: a mathematical model.. Proc R Soc A.

[21] Charlwood JD, Smith T, Billingsley PF, Takken W, Lyimo EOK (1997). Survival and infection probabilities of anthropophagic anophelines from an area of high prevalence of *Plasmodium falciparum* in humans.. Bull Entomol Res.

[22] Killeen GF, McKenzie FE, Foy BD, Schieffelin C, Billingsley PF (2000). A simplified model for predicting malaria entomologic inoculation rates based on entomologic and parasitologic parameters relevant to control.. Am J Trop Med Hyg.

[23] Anderson RA, Knols BGJ, Koella JC (2000). *Plasmodium falciparum* sporozoites increase feeding-associated mortality of their mosquito hosts *Anopheles gambiae* s.l.. Parasitology.

[24] Day JF, Edman JD (1983). Malaria renders mice susceptible to mosquito feeding when gametocytes are most infective.. J Parasitol.

[25] Rowland M, Hemingway J (1987). Changes in malathion resistance with age in *Anopheles stephensi* from Pakistan.. Pestic Biochem Physiol.

[26] Hodjati MH, Curtis CF (1999). Evaluation of the effect of mosquito age and prior exposure to insecticide on pyrethroid tolerance in *Anopheles* mosquitoes (Diptera: Culicidae).. Bull Entomol Res.

[27] Hunt RH, Brooke BD, Pillay C, Koekemoer LL, Coetzee M (2005). Laboratory selection for and characteristics of pyrethroid resistance in the malaria vector *Anopheles funestus*.. Med Vet Entomol.

[28] Lines JD, Nassor NS (1991). DDT resistance in *Anopheles gambiae* declines with mosquito age.. Med Vet Entomol.

[29] Rajatileka S, Burhani J, Ranson H (2011). Mosquito age and susceptibility to insecticides.. Trans R Soc Trop Med Hyg.

[30] Matambo TS, Abdalla H, Brooke BD, Koekemoer LL, Mnzava A (2007). Insecticide resistance in the malarial mosquito *Anopheles arabiensis* and association with the *kdr* mutation.. Med Vet Entomol.

[31] Rivero A, Vezilier J, Weill M, Read AF, Gandon S (2010). Insecticide control of vector-borne diseases: when is insecticide resistance a problem?. PLoS Pathog.

[32] Ferguson HM, Rivero A, Read AF (2003). The influence of malaria parasite genetic diversity and anaemia on mosquito feeding and fecundity.. Parasitology.

[33] Molina-Cruz A, Dejong RJ, Charles B, Gupta L, Kumar S (2008). Reactive oxygen species modulate *Anopheles gambiae* immunity against bacteria and *Plasmodium*.. J Biol Chem.

[34] Ferguson HM, Mackinnon MJ, Chan BH, Read AF (2003). Mosquito mortality and the evolution of malaria virulence.. Evolution.

[35] Cohuet A, Osta MA, Morlais I, Awono-Ambene PH, Michel K (2006). *Anopheles* and *Plasmodium*: from laboratory models to natural systems in the field.. EMBO Rep.

[36] Tripet F, Aboagye-Antwi F, Hurd H (2008). Ecological immunology of mosquito-malaria interactions.. Trends Parasitol.

[37] Farenhorst M, Knols BG, Thomas MB, Howard AF, Takken W (2010). Synergy in efficacy of fungal entomopathogens and permethrin against West African insecticide-resistant *Anopheles gambiae* mosquitoes.. PLoS ONE.

[38] Paaijmans KP, Blanford S, Bell AS, Blanford JI, Read AF (2010). Influence of climate on malaria transmission depends on daily temperature variation.. Proc Natl Acad Sci U S A.

[39] Jefferson AV, Hensley L, Beier JC (1994). Sporogonic development of *Plasmodium yoelii* in five anopheline species.. J Parasitol.

[40] WHO (1998). Test procedures for insecticide resistance monitoring in malaria vectors, bio-efficacy and persistence of insecticide on treated surfaces..

[41] Carlson J, Suchman E, Buchatsky L (2006). Densoviruses for control and genetic manipulation of mosquitoes.. Adv Virus Res.

[42] Cook PE, McMeniman CJ, O'Neill SL (2008). Modifying insect population age structure to control vector-borne disease.. Adv Exp Med Biol.

[43] McMeniman CJ, Lane RV, Cass BN, Fong AWC, Sidhu M (2009). Stable introduction of a life-shortening *Wolbachia* infection into the mosquito *Aedes aegypti*.. Science.

[44] Lines JD (1988). Do agricultural insecticides select for insecticide resistance in mosquitoes? A look at the evidence.. Parasitol Today.

